# Regulation by partition predicts enhancer target selection in mammalian genomes

**DOI:** 10.64898/2026.10.23.761042

**Published:** 2026-10-23

**Authors:** Jordan Valgardson, Tomek Swigut, Joanna Wysocka

## Abstract

Enhancers regulate specific promoters across long genomic ranges, yet how they select among the many promoters within reach remains unresolved. Here we introduce SPIE-seq (Synthetic Perturbation by Inserted Enhancer), which pairs piggyBac-mediated enhancer insertion with an allele-specific readout of cis-regulatory effects on expression. Across 724 clonal lines carrying 29,954 mapped insertions, we detected enhancer-dependent expression changes at 18,440 enhancer-gene pairs. We found that enhancer influence declines with genomic distance, following similar average distance-decay scaling across all tested enhancers, though the shape of response curves differs between loci. Genomic context, not enhancer identity, dominates target choice: distinct enhancers inserted at the equivalent positions activate nearly the same targets, while the same enhancer can activate discordant promoters when inserted at different positions within the same locus. Responsive promoters are enriched for developmental features, but target choice is nonetheless competitive: even at loci with high promoter density, an enhancer primarily activates only one or two promoters. Promoter skipping is common and increases as competitors accumulate — a pattern incompatible with promoters responding independently based solely on their intrinsic features. We formalize this in the Regulation-by-Partition (RbP) model, in which an enhancer allocates its output among the promoters according to their genomic distance, contact with the enhancer, and intrinsic features. RbP predicts target choice accurately and transfers without retraining across species, cell types, and perturbation direction, outperforming existing enhancer–gene models, with the largest gains at long genomic ranges. Enhancer target choice is thus not dictated by enhancer or promoter attributes alone but emerges from the distance-weighted competition among promoters within the regulatory landscape.

